# ATF2 predicts poor prognosis and promotes malignant phenotypes in renal cell carcinoma

**DOI:** 10.1186/s13046-016-0383-2

**Published:** 2016-07-04

**Authors:** Deng-shuang Wu, Cheng Chen, Zhen-jie Wu, Bing Liu, Li Gao, Qing Yang, Wei Chen, Jun-ming Chen, Yi Bao, Le Qu, Lin-hui Wang

**Affiliations:** Department of Urology, Changzheng Hospital, Second Military Medical University, 415 Fengyang Road, Shanghai, 200003 China; Department of Medical Oncology, Jinling Hospital, Nanjing University Clinical School of Medicine, Nanjing, 210002 China; Department of Pathology, Changhai Hospital, Second Military Medical University, Shanghai, 200433 China; Department of Urology, Changhai Hospital, Second Military Medical University, Shanghai, 200433 China; Department of Urology, No. 203 Hospital of People’s Liberation Army, Qiqihaer, 161000 Heilongjiang China; Department of Urology, Henan Provincial Corps Hospital of Chinese People’s Armed Police Force, Zhengzhou, 450052 China; Department of Urology, Jinling Hospital, Nanjing University Clinical School of Medicine, 305 East Zhongshan Road, Nanjing, 210002 China

**Keywords:** Renal cell carcinoma, ATF2, Proliferation, Metastasis, Prognosis

## Abstract

**Background:**

Activating transcription factor 2 (ATF2) is a basic helix-loop-helix transcription factor, which has been shown to participate in the pathobiology of numerous cancers. However, the role of ATF2 in renal cell carcinoma (RCC) remains unclear.

**Methods:**

ATF2 knockdown and overexpression studies were performed in RCC cells to evaluate changes in cell viability, cell cycle, apoptosis, migration and invasion. Xenograft models were used to examine the tumorigenic and metastatic capability of RCC cells upon ATF2 suppression. The expression of ATF2 in human RCC samples was determined using immunohistochemistry on a tissue microarray.

**Results:**

ATF2 knockdown in RCC cells reduced their proliferative and metastatic potentials, whereas ATF2 overexpression enhanced these properties. Mechanistic studies revealed that the transcription of CyclinB1, CyclinD1, Snail and Vimentin was directly regulated by ATF2 in RCC cells. Moreover, ATF2 was shown to be highly expressed in RCC tissues, especially in tumors with metastases. High expression of ATF2 correlated with aggressive clinico-pathological characteristics and predicted poor prognosis of RCC patients.

**Conclusions:**

ATF2 exerts an oncogenic role in RCC and could serve as an important prognostic biomarker.

**Electronic supplementary material:**

The online version of this article (doi:10.1186/s13046-016-0383-2) contains supplementary material, which is available to authorized users.

## Background

Renal cell carcinoma (RCC) is the most prevalent malignancy of adult kidney, and its incidence has been increasing in recent decades [1, 2]. Patients with early-stage disease can be effectively treated with surgical resection, however, approximately 20 % of patients present with metastatic disease at the time of initial diagnosis. Moreover, up to 20 % of RCC patients will develop metastases lesions following nephrectomy [3, 4]. Therefore, there is an urgent need to elucidate the molecular mechanisms underlying RCC progression, and identify novel biomarkers and therapeutic targets to improve the prognosis of RCC patients.

Activating transcription factor 2 (ATF2) is a member of ATF family [5], characterized by a basic structural region and a leucine zipper domain [6]. In response to extracellular stresses or inflammatory cytokines, the transcriptional activity of ATF2 is induced through Thr69- and/or Thr71- phosphorylation by Jun N-terminal kinase (JNK) or p38 (MAPK14) [7–9]. In melanoma and non-small cell lung carcinoma, ATF2 acts as an important oncogene [10, 11], while in nonmalignant skin and breast cancer, ATF2 elicits tumor-suppressor function [12, 13], suggesting a context-dependent role for ATF2 in cancer biology.

Currently, the role of ATF2 in the physiology and pathology of kidney is limited to the evidence that ATF2 has been demonstrated to play a key role in the morphogenesis and apoptosis of renal epithelial cells [14, 15], possibly resulting in renal cyst formation [16]. However, the role of ATF2 in RCC remains unknown. Herein, we examined the effects of ATF2 on the malignant phenotypes of RCC cells, and explored its clinical significance in patients with RCC.

## Methods

### Cell lines and reagents

The human RCC cell lines ACHN and 786-O were purchased from American Type Culture Collection (ATCC, Rockville, MD, USA) and were cultured in MEM (Invitrogen, Carlsbad, CA, USA) and RPMI-1640 (Gibco, Thermo Fisher Scientific, Waltham, MA, USA) respectively, and supplemented with 10 % fetal bovine serum (Gibco, USA). Matrigel was purchased from BD (USA).

### Patients and clinical samples

RCC patients who underwent surgery between January 2007 and April 2014 at the Department of Urology, Changzheng Hospital, Second Military Medical University (Shanghai, China) were enrolled in our study (*n* = 205). The ethical approval was granted from the Committees for Ethical Review at Second Military Medical University and informed consent was obtained from all patients. All specimens were formalin-fixed, paraffin embedded and pathologically confirmed, and subsequently used to construct a tissue microarray. The expression levels of ATF2 were examined by immunohistochemistry.

### Immunohistochemistry (IHC)

Paraffin embedded sections were deparaffinized, rehydrated, and then prepared for antigen retrieval. Sections were blocked with 10 % goat serum and incubated with primary antibody against ATF2 (Proteintech-14834-1-AP, dilution 1:50) at 4 °C overnight, and biotin-labeled secondary antibody for 1 h at room temperature. Subsequently, the samples were developed by adding DAB and counterstained with hematoxylin (Beyorime Institute of Biotechnology, Inc.).

### Plasmids and cell transfection

Human full-length cDNA of ATF2 was cloned into expression plasmid pCMV3-ATF2-Flag (Sino Biological lnc.). A short hairpin RNA (shRNA) sequence was designed by Hanbio Biotechnology Co. Ltd (Shanghai, China) to target human ATF2 gene (NM_001256090.1). After annealing, double strands of shRNA were inserted into lentiviral pHBLV-U6-Puro vector (Addgene). For lentiviral packaging, HEK-293 T cells were co-transfected with the lentiviral vector, and packaging vectors psPAX2 and PMD2G using LipoFiter™ Liposomal Transfection Reagent (Hanbio, Shanghai, China), according to the manufacturer instructions. RCC cells were seeded in 6-well plates and grown to 50 % confluence on the day of infection. Four hours prior to infection, cells were placed in serum-free media and lentivirus particles were added to the culture medium at a multiplicity of infection (MOI) of 30. Cells were grown at 37 °C, and 24-h following transfection cells were placed with fresh media. The sequences of primers used for plasmid construction in this study were provided in Additional file 1: Table S1.

Transfections were carried out using jetPRIME® - Polyplus-transfection according to the manufacturer’s protocol. One day prior to transfection, cells were seeded in six-well plates. 4 μg of DNA was diluted into 150 mM NaCl and 6 μl of jetPEI® was diluted into 150 mM NaCl to a final volume of 200 μl per well. After 12 h, cells were placed in fresh media.

### RNA extraction and quantitative real-time PCR (qRT-PCR)

Total RNA was extracted from cells and tissues using TRIzol reagent (Invitrogen) according to the manufacturer instructions. First-strand cDNA was generated from 2 μg total RNA using the PrimeScript RT reagent kit (Takara, Dalian, China) with random primers. qRT-PCR was performed on an ABI Prism 7300 (Applied Biosystems, Foster City, CA, USA). Sequences of primers used for qRT-PCR in this study were provided in Additional file 1: Table S2. The relative expression level of indicated genes was compared to that of β-Actin and was calculated using the 2^−ΔΔCt^ method. Each qRT-PCR reaction was performed in triplicate.

### Western blot analysis

RCC cells were lysed using 1 × SDS sample buffer and heated at 95 °C for 10 min. Quantified proteins were separated on an SDS-PAGE and transferred to PVDF membranes. The membranes were blocked with 5 % nonfat milk for 2 h at room temperature and incubated with specific antibodies. The following primary antibodies were used in our study: ATF2 (Proteintech-14834-1-AP), Phospho-ATF2 (Thr71) (CST-9221), CyclinB1 (CST-12231), CyclinD1 (CST-2978), E-Cadherin (BS-1097), Snail (BS-1853), Vimentin (BS-1491) and β-Actin (Santa cruz-81178). Band intensity was quantitatively analyzed using Quantity One software (Bio-Rad, USA), and the absolute intensity of target protein was normalized to the absolute intensity of β-Actin.

### Cell proliferation assay

Cell proliferation was analyzed using the Cell Counting Kit 8 (CCK8, Beyotime Institute of Biotechnology, Shanghai, China). RCC cells were seeded in 96-well plates at a density of 2000 cells per well. At indicated time points, 10 μl CCK8 solution was added into each well and incubated for 2 h. The absorbance at 450 nm was measured to assess the number of viable cells. The results were obtained from three independent experiments in triplicate.

### Plate colony formation assay

ACHN cells (500 cells) were seeded into 10 cm plates and cultured in the 37 °C incubator for ~10 days until most single colony were composed of more than 100 cells. The plates were washed by PBS, fixed with 4 % paraformaldehyde, and stained with crystal violet. The number of colonies containing more than 100 cells was counted in each well.

### Flow cytometry analysis

Cell apoptosis and cell cycle progression were quantified using flow cytometry analysis (BD Biosciences, San Jose, CA). For apoptosis experiment, RCC cells were collected and washed twice with ice-cold PBS and re-suspended in 200 μl binding buffer. FITC-conjugated Annexin V was added to a final concentration of 0.5 μg/ml and incubated for 20 min at room temperature in the dark, prior to the addition of 1 μg/ml propidium iodide (PI). Samples were immediately analyzed by flow cytometry. For cell cycle progression, RCC cells were collected, washed with PBS and fixed with 75 % ethanol. Cells were stained with PI and RNase overnight at 4 °C. Samples were analyzed by flow cytometry.

### Wound-healing assay

RCC cells were seeded at 5 × 10^5^ cells/well in 6-well plates and cultured until the plates were confluent. The cell monolayer was scraped in a straight line using a 10 μl pipette tip to create a scratch, washed with PBS twice and the medium was replaced with serum free medium. Images were captured at 0, 6, and12 h following the initial scratch to evaluate cell migration.

### Transwell assay

Cell transwell assays were performed with 24-well transwell chamber uncoated (migration) or matrigel-coated (invasion) according to the manufacturer instructions (pore size 8 μm, Corning Life Sciences, NY, USA). 1 × 10^4^ serum pre-starved RCC cells in 250 μl serum free media were seeded into the upper chamber, and the bottom chamber contained medium supplemented with 10 % FBS. After 24 h incubation, the cells on the upper surface of the membrane were scraped off, and the cells on the bottom side of the membrane were fixed with 4 % paraformaldehyde and stained with crystal violet. Cells were counted from 8 randomly chosen fields (magnification, ×200).

### Chromatin immunoprecipitation (ChIP)

Following transfection, 786-O cells (1 × 10^7^ cells) were cross-linked with 1 % formaldehyde and incubated for 10 min at 37 °C. ChIP assay was performed according to the manufacturer’s protocol (Millipore, USA) using monoclonal Anti-Flag® M2 antibody produced in mouse (Sigma) or normal rabbit IgG as a negative control (Santa Cruz Biotechnology). An aliquot of lysates (20 μl) was taken out as input control. DNA enrichment was determined by quantitative PCR (qPCR), and was normalized to input. Sequences of primers used for ChIP-qPCR in this study were provided in Additional file 1: Table S3. The products of qPCR were detected by agarose gel electrophoresis.

### In vivo tumor xenograft experiment

Male nude mice (BALB/c Nude; 4 weeks old) were purchased from the Shanghai Institute of Material Medical (Chinese Academy of Science) and maintained in a pathogen-free condition in accordance with relevant guidelines and regulations for the care and use of laboratory animals, with the approval of the Institutional Animal Care and Use Committee at Second Military Medical University. ACHN cells (5 × 10^6^ cells) in 100 μl PBS were implanted subcutaneously into the flanks of nude mice. Tumor size was monitored at 3 days intervals using calipers, and the tumor volumes were calculated according to the following formula: tumor volume = largest diameter × perpendicular height^2^ × 0.5. For lung metastasis model, ACHN cells (1 × 10^6^ cells) in 200 μl PBS were injected into the tail vein of mice. Mice were sacrificed 12 weeks after inoculation and consecutive sections of the whole lung were subjected to hematoxylin-eosin staining. All of the metastatic lesions in lung were calculated microscopically to evaluate the development of pulmonary metastasis.

### TUNEL assay

Apoptosis in tumor tissues was detected by DNA fragmentation with an apoptosis detection kit (Roche TUNEL-Apo-AP; USA) according to the manufacturer instruction. Tissues were deparaffinized and hydrated as previously described. The samples were developed by adding DAB and counterstained with hematoxylin (Beyorime Institute of Biotechnology, Inc.). Images were taken with a Nikon microscope at 200× magnifications.

### Statistical analysis

Statistical analysis was performed with SPSS Statistics software version 19 (SPSS Inc., USA). Data were presented as “mean ± SD”. Pearson chi-square test was used to analyze the clinical variables. Kaplan-Meier method and log-rank tests were used to compare RCC patient survival based on dichotomized ATF2 expression. Cox proportional hazards regression analysis were used to analyze the independent factors on the survival prognosis of patients with RCC. Differences between groups were analyzed by Student’s t-test after evaluating the normal distribution of results. A p-value < 0.05 was considered statistically significant.

## Results

### ATF2 is highly expressed in RCC tissues

**Fig. 1:**
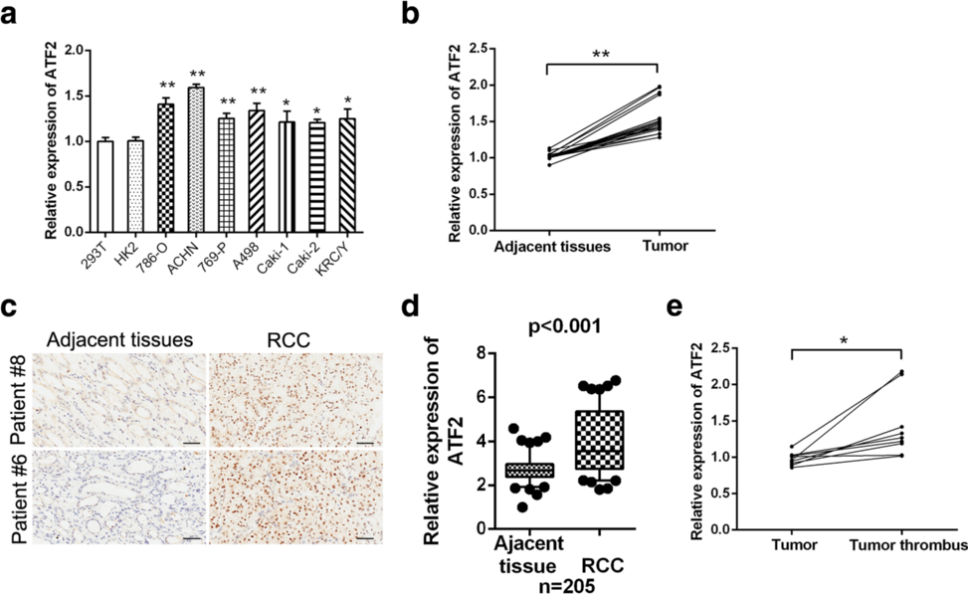
ATF2 is highly expressed in RCC samples. **a** qRT-PCR analysis of ATF2 in RCC cells versus human normal renal cells (293 T and HK-2). **b** qRT-PCR analysis of ATF2 in RCC tissues and adjacent non-tumor tissues from 17 RCC patients. **c** Immunohistochemistry analysis of ATF2 in RCC tissues (*right*) and adjacent non-tumor tissues (*left*). Scale bar = 50 μm. **d** Immunohistochemical comparison of ATF2 expression in human RCC tissues versus adjacent non-tumor tissues. The horizontal lines in the box plots represent the median, the boxes represent the interquartile range, and the whiskers represent the 2.5th and 97.5th percentiles. **e** qRT-PCR analysis of ATF2 in primary RCC tissues and tumor thrombus tissues from 9 patients. Results are presented as mean ± SEM from three independent experiments. *p < 0.05 and **p <0.01

The expression of ATF2 was detected in various RCC cell lines. As shown in Fig. 1a, the upregulation of ATF2 was observed in RCC cell lines compared to normal kidney cell lines. The average mRNA expression of ATF2 was higher in RCC tumors than that in corresponding adjacent normal tissues (Fig. 1b). Immunostaining analysis on tissues microarray further confirmed that ATF2 was increased in RCC tissues and showed predominant expression in nucleus compared with adjacent normal tissues (Fig. 1c and Fig. 1d). Notably, ATF2 expression was even higher in tumor thrombus than primary tumors (Fig. 1e). Together, these results prompted an oncogenic role of ATF2 in RCC progression.

### ATF2 promotes RCC cells proliferation by promoting Cyclin expression

**Fig. 2:**
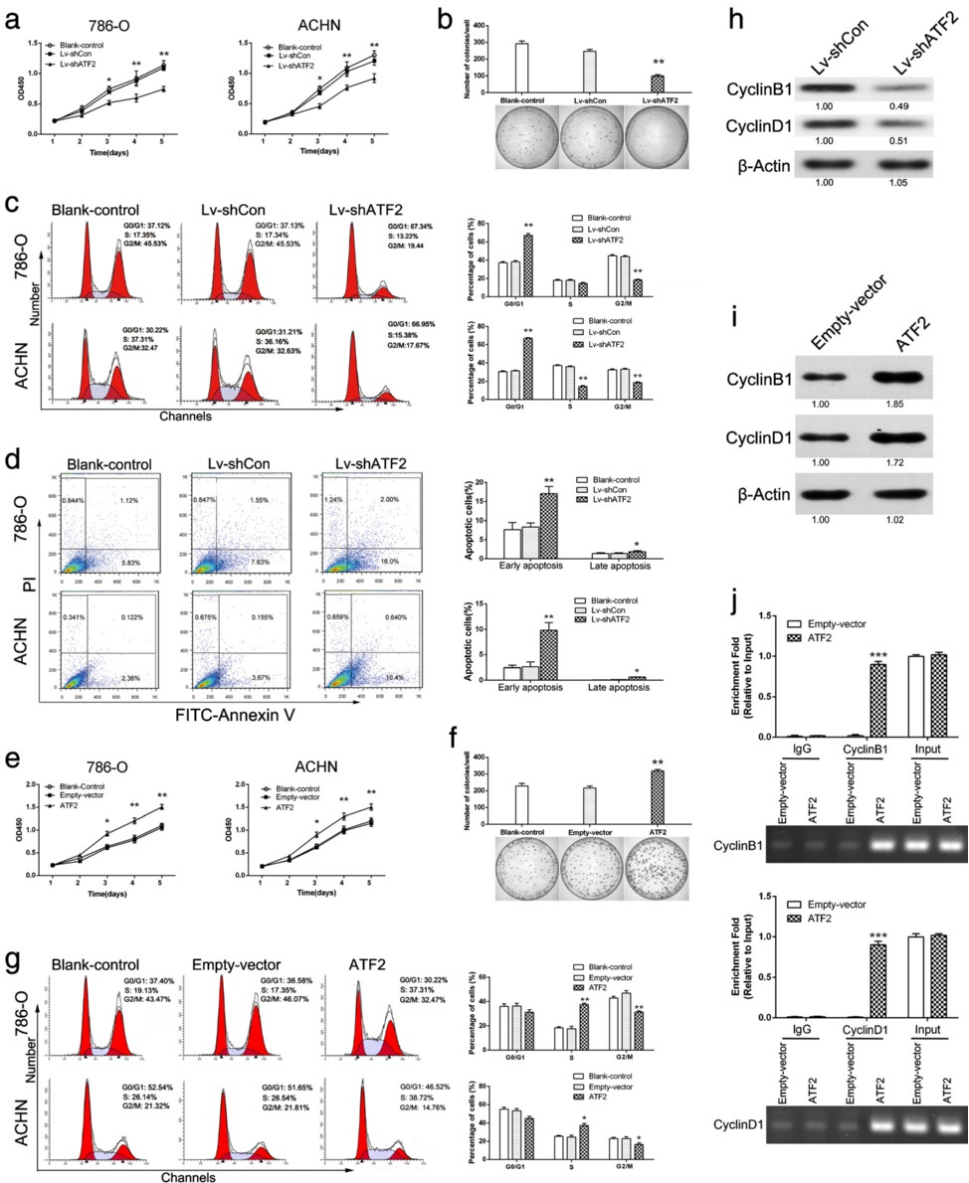
ATF2 promotes RCC cells proliferation in vitro. **a** CCK8 assay of ATF2 knockdown and control RCC cells at indicated times. **b** Plate colony formation assay of ATF2 knockdown and control ACHN cells in 10 cm dish for 3 weeks (*n* = 3). Average number of colonies (*upper*) and representative images (*lower*) were shown. **c** Flow cytometry analysis of cell cycle in ATF2 knockdown and control RCC cells. Representative cell cycle distributions were shown (*left*) and the histogram columns represent the average percentages of G0/G1, S and G2/M phases (*right*). **d** Flow cytometry analysis of apoptotic cells in ATF2 knockdown and control RCC cells (*left*) and the percentage of cells at the different apoptosis phases (*right*). The bar charts showed the increases in the early and late apoptotic indexes of RCC cells transfected with shATF2. **e** CCK8 assay of ATF2 overexpression and control RCC cells at indicated times. **f** Plate colony formation assay of ATF2 overexpression and control ACHN cells in 10 cm dish for 3 weeks (*n* = 3). Average number of colonies (*upper*) and representative images (*lower*) were shown. **g** Flow cytometry analysis of cell cycle in ATF2 overexpression and control RCC cells. Representative cell cycle distributions were shown (*left*) and the histogram columns represent the average percentages of G0/G1, S and G2/M phases (*right*). **h** & **i** Western blotting analysis of Cyclin B1 and Cyclin D1 in ATF2 knockdown (**h**), ATF2 overexpression (**i**) and control ACHN cells. β-Actin was used as an internal standard. **j** ChIP assay analysis of the enrichment of ATF2 at the proximal promoter region of Cyclin B1 and Cyclin D1 in the context of ATF2 overexpression. The enrichment of ATF2 (*upper*) on Cyclin B1 or Cyclin D1 promoter relative to input in 786-O cells and gel electrophoresis of PCR products from ChIP assay (*lower*). Results are presented as mean ± SEM from three independent experiments. *p < 0.05, **p <0.01 and *** *p* < 0.001

To investigate the biological function of ATF2 in RCC cells, lentivirus-based short hairpin RNAs (shRNAs) was used to suppress ATF2 expression. ATF2 shRNA substantially decreased ATF2 mRNA and protein levels compared to control groups (Additional file 1: Figure S1A and 1B). CCK8 assay and plate colony formation assay were performed to characterize the role of ATF2 in cell proliferation. As shown in Fig. 2a, ATF2 knockdown resulted in reduced proliferation of RCC cells. The size and number of single colony was also decreased in ATF2 knockdown ACHN cells (Fig. 2b).

We then evaluated the cell cycle distribution by flow cytometry. Knockdown of ATF2 increased the proportion of cells in G0/G1 phase, with concomitant decrease in S and G2/M phase (Fig. 2c). These results indicated that ATF2 may promote cell cycle progression. In addition, suppression of ATF2 enhanced the ratio of early and late apoptotic cells (Fig. 2d).

Conversely, exogenous expression of ATF2 in RCC cells (Additional file 1: Figure S1C and 1D) enhanced cell proliferation and induced an increase in the number of cells in S phase compared to control cells (Fig. 2e-2g).

Cell cycle is precisely regulated by cyclins, we therefore evaluated the effect of ATF2 on their expressions. Notably, the mRNA and protein expression of CyclinB1 and CyclinD1 were downregulated upon ATF2 knockdown (Additional file 1: Figure S2A and Fig. 2h) and upregulated upon ATF2 overexpression (Additional file 1: Figure S2B and Fig. 2i). Moreover, ChIP assay demonstrated that ATF2 was enriched on the proximal promoters of CyclinB1 and CyclinD1 in cells overexpressing ATF2 (Fig. 2j), indicating that ATF2 bound to the promoter of CyclinB1 and CyclinD1 and promoted their transcription. Taken together, these results revealed that ATF2 promotes RCC cells proliferation at least in part by transactivating the expression of CyclinB1 and CyclinD1.

### ATF2 facilitates RCC cell migration and invasion

**Fig. 3:**
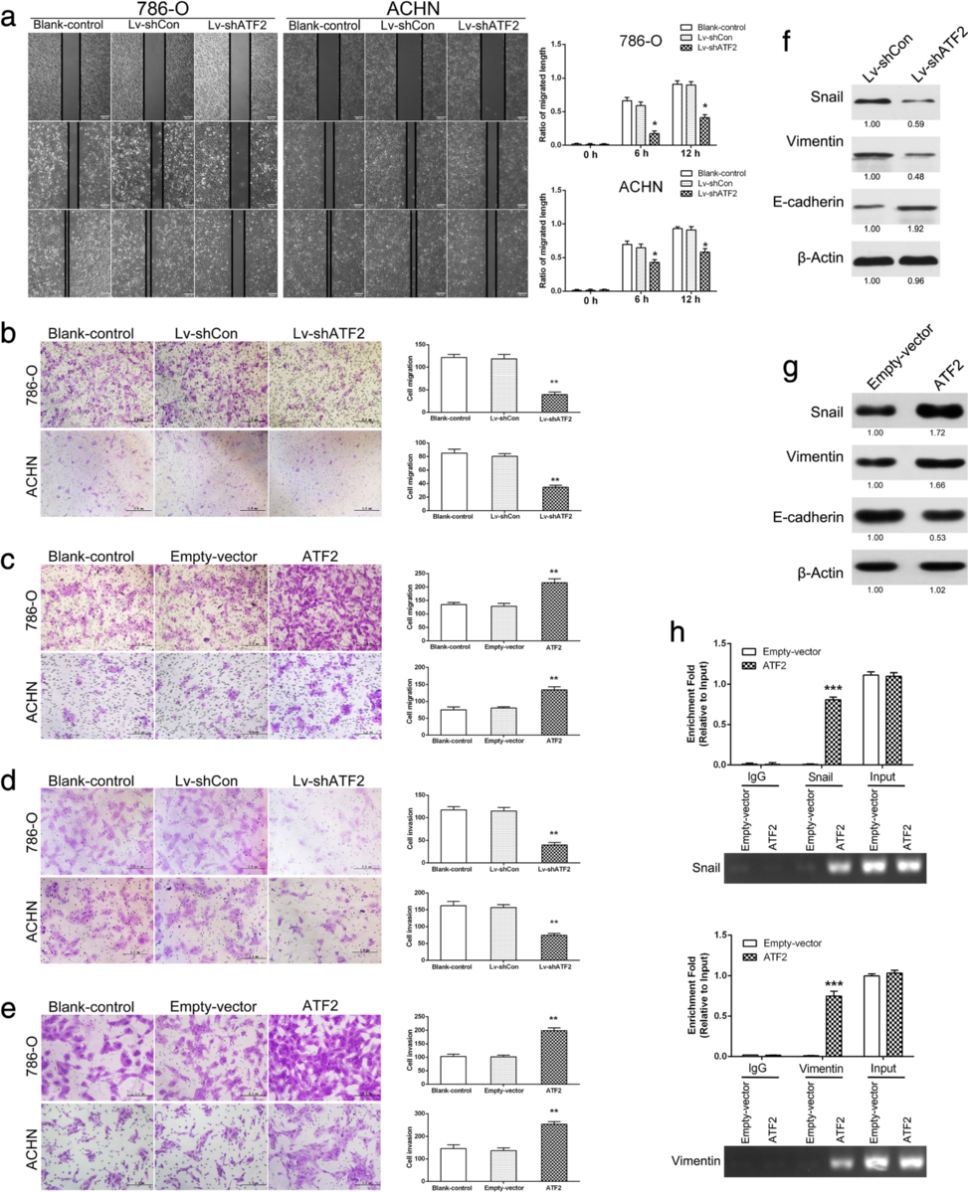
ATF2 facilitates RCC cell migration and invasion in vitro. **a** Left: representative images of the wound-healing assay of ATF2 knockdown and control RCC cells photographed at 0, 6 and 12 h after scratching. Scale bar = 200 μm. Right: the relative migration rate was calculated by dividing the change in the distance between the scratch edges by the initial distance. **b** & **c** Transwell assays were performed to evaluate cell migration following ATF2 knockdown (**b**), ATF2 overexpression (**c**) and control RCC cells. The statistical graph indicates the means ± SEM of the number of cells from 8 random high power fields (magnification, ×200) counted from three independent experiments. Scale bar = 2 mm. **d** & **e** Transwell assays were performed to evaluate the invasion of ATF2 knockdown (**d**), ATF2 overexpression (**e**) and control RCC cells. The statistical graph indicates the means ± SEM of the number of cells from 8 random high power fields (magnification, ×200) counted from three independent experiments. Scale bar = 2 mm. **f** & **g** Western blotting analysis of Snail, Vimentin and E-Cadherin in ATF2 knockdown (**f**), ATF2 overexpression (**g**) and control 786-O cells. β-Actin was used as an internal standard. **h** ChIP assay analysis of the enrichment of ATF2 at the proximal promoter region of Snail and Vimentin in the context of ATF2 overexpression. The enrichment of ATF2 on Snail and Vimentin promoter relative to input in 786-O cells (upper). The gel electrophoresis of PCR products from ChIP assay (lower). Results are presented as mean ± SEM from three independent experiments. *p < 0.05, **p <0.01, and ***p < 0.001

RCC is prone to metastasis to distant organs [17]. We next explored the role of ATF2 in cell migration and invasion. Wound-healing assay and transwell assay revealed that ATF2 knockdown significantly inhibited cell migration (Fig. 3a and 3b), while ATF2 overexpression enhanced the migratory abilities of RCC cells (Fig. 3c). Matrigel invasion chamber assay demonstrated that ATF2 knockdown suppressed cell invasion in RCC cells (Fig. 3d) and ATF2 overexpression led to enhanced invasion (Fig. 3e).

Epithelial to mesenchymal transition (EMT) plays a pivotal role in tumor metastasis [18, 19]. We thus examined the effect of ATF2 on the expression of EMT markers. Increased levels of epithelial markers (E-Cadherin) and decreased levels of mesenchymal markers (Snail and Vimentin) were detected in RCC cells following ATF2 knockdown (Additional file 1: Figure S2C and Fig. 3f). Conversely, ATF2 overexpression decreased E-Cadherin expression and increased the expression of Snail and Vimentin (Additional file 1: Figure S2D and Fig. 3g). Furthermore, we observed an enrichment of ATF2 at the proximal promoter region of Snail and Vimentin upon ATF2 overexpression (Fig. 3h). Thus, these results suggest that ATF2 promotes RCC metastasis partially depending on EMT induction.

### ATF2 knockdown suppresses tumor growth and metastasis in vivo

**Fig. 4:**
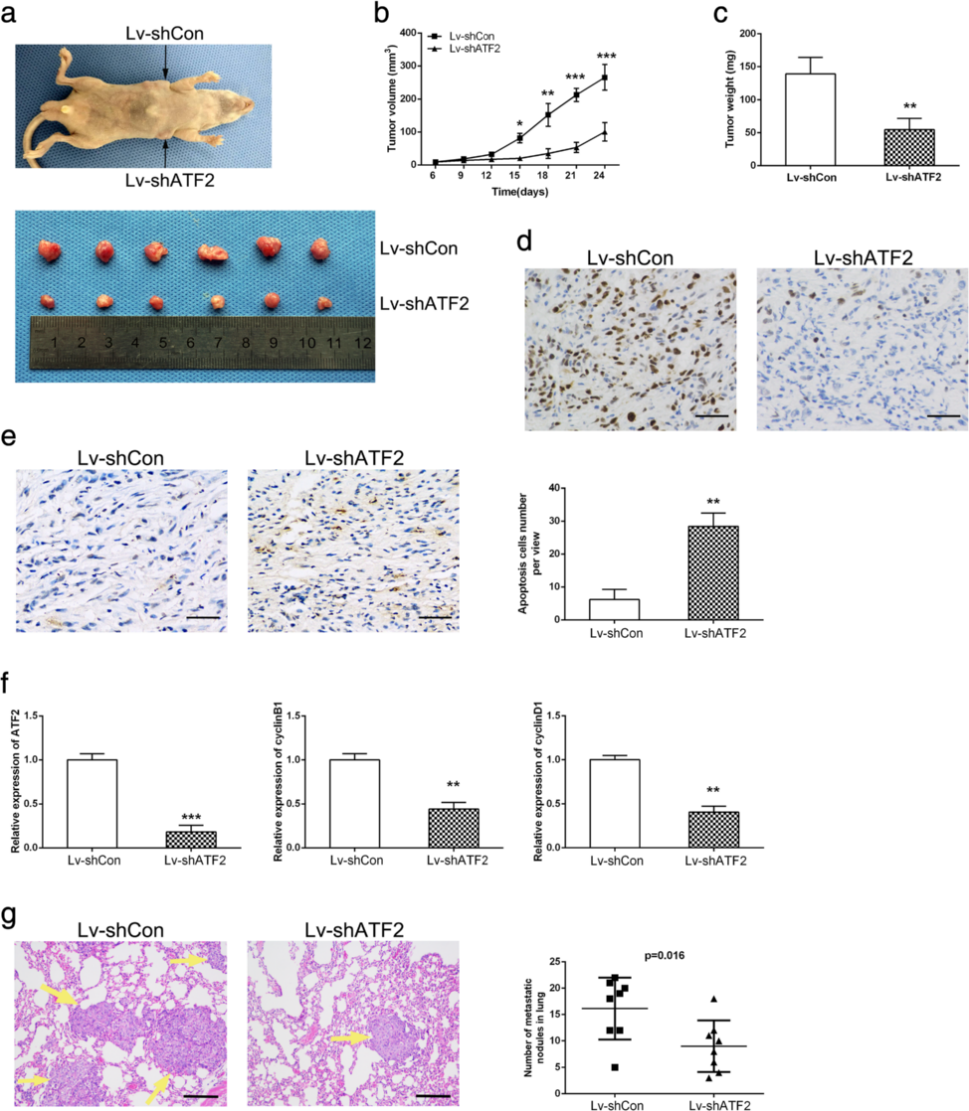
ATF2 knockdown suppresses tumor growth in vivo. **a** A representative nude mice showing the morphology of the tumors derived from ATF2 knockdown and control ACHN cells (upper). The tumors were dissected and photographed (lower). **b** The growth curve of the ATF2 knockdown versus the control ACHN tumors. **c** The average weight of ATF2 knockdown versus the control ACHN tumors. **d** Ki-67 staining of ATF2 knockdown versus the control ACHN tumors. Scale bar = 50 μm. **e** TUNEL assay analysis of the cell apoptosis in ATF2 knockdown versus the control ACHN tumors (left). Apoptosis cells numbers per view were shown (right). Scale bar = 50 μm. **f** qRT-PCR analysis of ATF2, Cyclin B1 and Cyclin D1 in ATF2 knockdown versus the control ACHN tumors. **g** Left: representative images of HE staining of metastatic nodules in the lungs of nude mice. The metastatic nodules are indicated by yellow arrows. Scale bar = 100 μm. Right: the numbers of nude mice with metastatic nodules in the lungs were calculated and compared (*p* = 0.016). Results are presented as means ± SEM for each group (*n* = 6). *p < 0.05, **p <0.01, and ***p < 0.001

To further explore the role of ATF2 in RCC growth in vivo, ATF2 knockdown or control ACHN cells were injected subcutaneously into nude mice. As shown in Fig. 4a-4c, tumor volume and weight were reduced in ATF2 shRNA group compared to control group. Immunostaining showed the proliferation marker Ki-67 was weaker in ATF2 knockdown tumors (Fig. 4d). And TUNEL assay demonstrated that apoptotic cells were increased in tumors from ATF2 shRNA group (Fig. 4e). In addition, the mRNA levels of CyclinB1 and CyclinD1 were decreased in the ATF2 knockdown tumors (Fig. 4f). These data demonstrate that the inhibition of ATF2 suppressed the tumorigenicity of RCC cells in vivo.

To examine the effect of ATF2 on RCC metastasis in vivo, we established a lung metastasis model. As shown in Fig. 4g, RCC cells with ATF2 knockdown formed decreased number and size of pulmonary metastatic lesions in mice.

### High levels of ATF2 predict poor prognosis of RCC patients

**Fig. 5:**
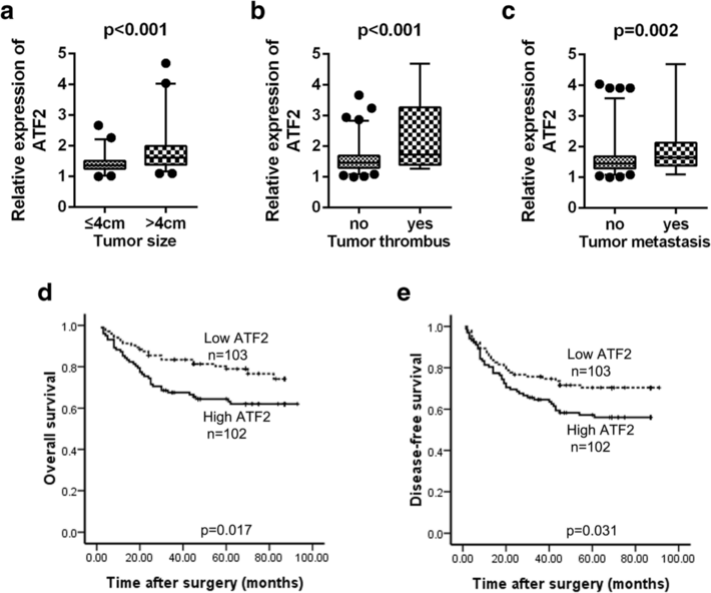
High levels of ATF2 predicts poor prognosis of RCC patients. **a** & **b** & **c** Comparison of ATF2 expression in human RCC tissues with diameter > 4 cm or ≤ 4 cm (**a**), RCC tissues with or without tumor thrombus (**b**), RCC tissues with or without tumor metastasis (**c**) determined by immunohistochemistry. The horizontal lines in the box plots represent the median, the boxes represent the interquartile range, and the whiskers represent the 2.5th and 97.5th percentiles. **d** Patients in comparative ATF2 high group (*n* = 102) had lower overall survival time than those in comparative ATF2 low group (*n* = 103) (*P* = 0.017). **e** Patients in comparative ATF2 high group (*n* = 102) had lower disease-free survival time than those in comparative ATF2 low group (*n* = 103) (*P* = 0.031)

To evaluate the clinical significance of ATF2 in RCC patients, we analyzed the correlation between ATF2 expression and the clinical characteristics of patients. As shown in Fig. 5a-5c, ATF2 was elevated in RCC tissues with larger tumor size, tumor thrombus or distant metastasis. According to the median expression of ATF2, 205 RCC patients were divided into two groups: high-ATF2 (*n* = 102) and low-ATF2 (*n* = 103). High ATF2 expression was associated with larger tumor size (*p* = 0.007), advanced pathological stage (*p* = 0.024), tumor thrombus (*p* = 0.032) and distant metastasis (*p* = 0.022) (Additional file 1: Table S4). Furthermore, patients in the high ATF2 group exhibited a worse overall survival (OS) and disease-free survival (DFS) than those in low ATF2 group (Fig. 5d and 5e). Multivariate analysis identified high ATF2 level in RCC tissues as an independent prognostic factor for RCC patients (Additional file 1: Tables S5 and S6).

## Discussion

Emerging evidence has indicated that ATF2 exerts paradoxical roles in different cancers [20]. For instance, ATF2 acts as a tumor suppressor in breast cancer by promoting the expression of the apoptosis-regulated gene GADD45α and suppressor gene Maspin [21]. Similarly, the deletion of ATF2 in keratinocytes upregulated of β-catenin and downregulated of Notch consequently facilitated skin tumor formation [13]. By contrast, inhibition of ATF2 repressed tumorigenesis of melanoma via increasing JNK/Jun and JunD activities [22]. Therefore, the role of ATF2 may depend on cancer-specific contexts and its role in RCC remains unknown. In this study, we reported the oncogenic role of ATF2 in the regulation of RCC growth and metastasis both in vitro and in vivo. By using a large cohort of RCC patients, we identified ATF2 as a novel biomarker to predict RCC prognosis.

Cell proliferation needs cell cycle progression, which is known to be controlled by cyclins. Cyclin B1 and Cyclin D1 are key regulators for G1/S and G2/M transition respectively [23–26], depletion of which inhibited proliferation and induced apoptosis in many human tumors [23]. ATF2 has been reported to modify Cyclin B1 and Cyclin D1 levels to promote cell-cycle progression in several types of neoplasms, including cervical and breast cancer [27, 28]. Our results further revealed the enrichment of ATF2 at the proximal promoter region of Cyclin B1 and Cyclin D1, suggesting that ATF2 promotes the transcription of Cyclin B1 and Cyclin D1 to enhance RCC cell proliferation.

EMT plays a pivotal role in tumor metastasis and contributes to early-stage dissemination of cancer cells [29–31]. ATF2 was reported to promote metastasis by regulating EMT in solid tumors such as pancreatic cancer [32]. However, this relationship has never been demonstrated in RCC. Herein, we found that depletion of ATF2 caused a conversion from mesenchymal to epithelial phenotype, marked by upregulation of E-cadherin and downregulation of Snail and Vimentin. Furthermore, we found that Snail and Vimentin were direct downstream targets of ATF2 in RCC cells, suggesting that ATF2 enhanced metastasis of RCC cells by the induction of EMT.

As a transcription factor, the divergent roles of ATF2 may be associated with differential subcellular localization [33]. In the present study, we found that ATF2 expression was higher in RCC samples and primarily localized in the nucleus of RCC cells (Fig. 1c), suggesting the transcriptional function of ATF2. Consistent with the oncogenic role of ATF2 in functional study, RCC patients with high ATF2 expression were associated with aggressive clinico-pathological characteristics. Moreover, high ATF2 level was significantly correlated with decreased survival in RCC patients, and could serve as an independent predictor for poor prognosis.

## Conclusions

In summary, ATF2 plays a critical role in promoting cell proliferation and metastasis of RCC, and could serve as an independent predictor for the clinical outcome in RCC patients. Based on these findings, targeting ATF2 may represent a potential therapeutic strategy to curb the progression of RCC.

## Abbreviations

ATF2, activating transcription factor 2; CCK8, cell counting kit 8; ChIP, chromatin immunoprecipitation; DFS, disease free survival; EMT, epithelial to mesenchymal transition; IHC, immunohistochemistry; JNK, Jun N-terminal kinase; MOI, multiplicity of infection; OS, overall survival; PI, propidium iodide; qPCR, quantitative PCR; qRT-PCR, quantitative real-time PCR; RCC, renal cell carcinoma; shRNA, short hairpin RNA

## Additional files

Additional file 1: Supplemental **Figure S1.** The confirmation of ATF2 knockdown and overexpression. Supplemental **Figure S2.** qRT-PCR analysis of indicated genes expression upon ATF2 knockdown and overexpression. Supplemental **Table S1.** Sequences of primers used for plasmid construction. Supplemental **Table S2.** Sequences of primers used for qRT-PCR. Supplemental **Table S3.** Sequences of primers used for ChIP-qPCR. Supplemental **Table S4.** Correlation of ATF2 expression and clinical characteristics in RCC patients. Supplemental** Table S5.** Univariate and multivariate analyses of factors associated with overall survival in RCC patients. Supplemental **Table S6.** Univariate and multivariate analyses of factors associated with disease-free survival in RCC patients. (DOCX 625 kb)
