# Supplementary material for: ATF2 predicts poor prognosis and promotes malignant phenotypes in renal cell carcinoma
Source: J Exp Clin Cancer Res. 2016 Jul 4;35:108. doi: 10.1186/s13046-016-0383-2 (PMC4932740; doi:10.1186/s13046-016-0383-2)
Supplement: Additional file 1: — Supplemental Figure S1. The confirmation of ATF2 knockdown and overexpression. Supplemental Figure S2. qRT-PCR analysis of indicated genes expression upon ATF2 knockdown and overexpression. Supplemental Table S1. Sequences of primers used for plasmid construction. Supplemental Table S2. Sequences of primers used for qRT-PCR. Supplemental Table S3. Sequences of primers used for ChIP-qPCR. Supplemental Table S4. Correlation of ATF2 expression and clinical characteristics in RCC patients. Supplemental Table S5. Univariate and multivariate analyses of factors associated with overall survival in RCC patients. Supplemental Table S6. Univariate and multivariate analyses of factors associated with disease-free survival in RCC patients. (DOCX 625 kb) [file 13046_2016_383_MOESM1_ESM.docx]

**Supplemental Figure 1, related to Figure 2 and Figure 3.**

**
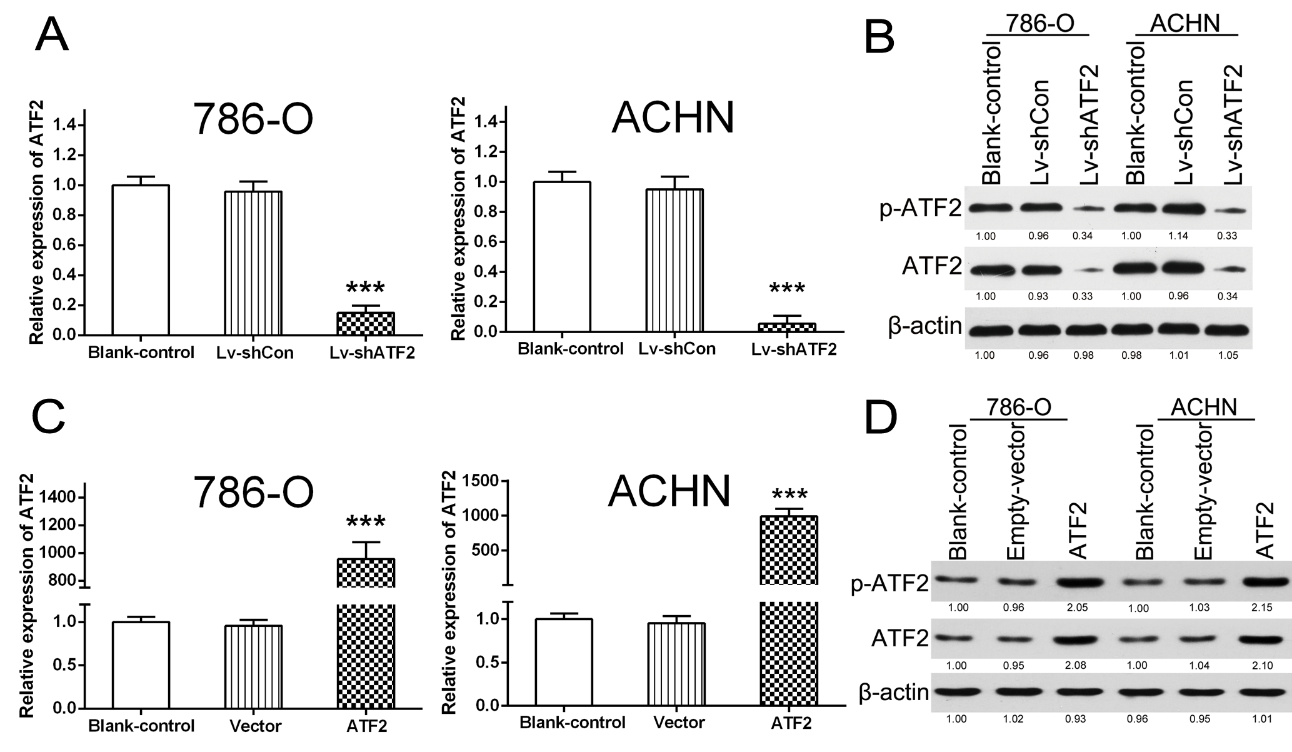
**

(A) qRT-PCR analysis of ATF2 in ATF2 knockdown and control RCC cells.

(B) Western blotting analysis of ATF2 in ATF2 knockdown and control RCC cells.

(C) qRT-PCR analysis of ATF2 in ATF2 overexpression and control RCC cells.

(D) Western blotting analysis of ATF2 in ATF2 overexpression and control RCC cells.

Results are presented as mean ± SEM from three independent experiments. ***p < 0.001.

**Supplemental Figure 2, related to Figure 2 and Figure 3.**

**
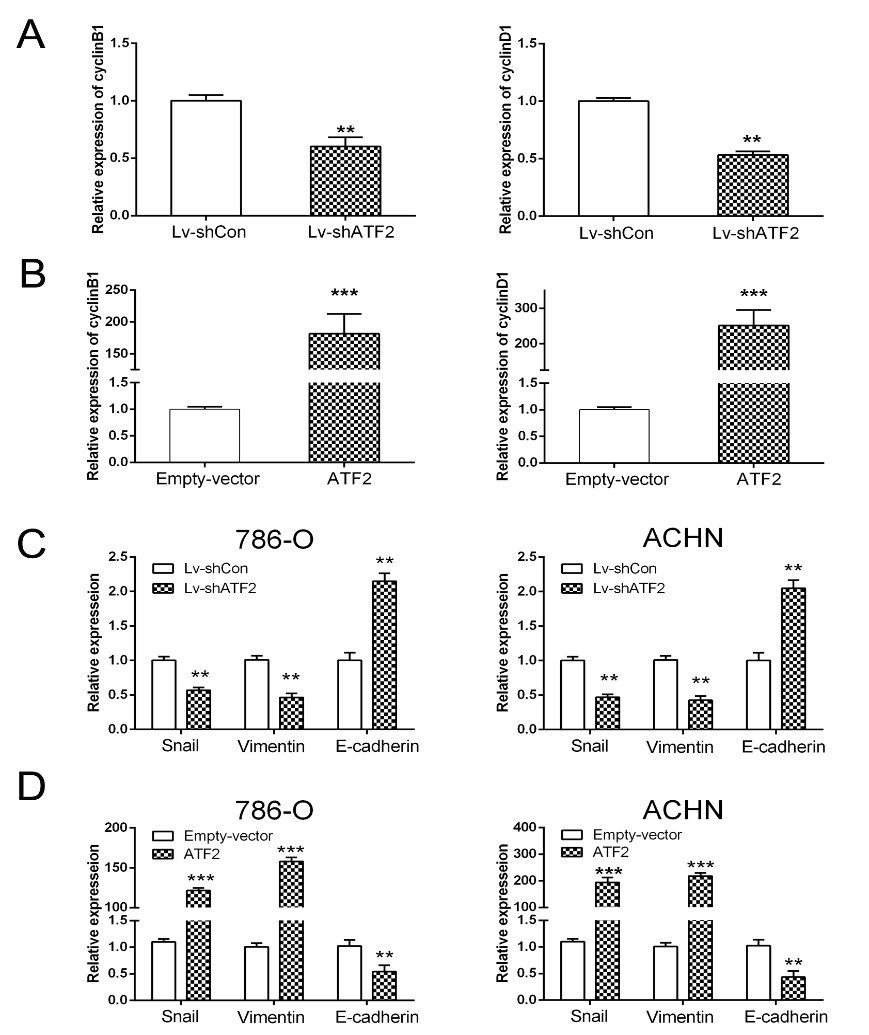
**

(A) qRT-PCR analysis of CyclinB1 and CyclinD1 in ATF2 knockdown and control ACHN cells.

(B) qRT-PCR analysis of CyclinB1 and CyclinD1 in ATF2 overexpression and control ACHN cells.

(C) qRT-PCR analysis of Snail, Vimentin and E-cadherin in ATF2 knockdown and control RCC cells.

(D) qRT-PCR analysis of Snail, Vimentin and E-cadherin in ATF2 overexpression and control RCC cells.

Results are presented as mean ± SEM from three independent experiments. **p< 0.01, ***p < 0.001.

**Supplemental Table 1. Sequences of primers used for plasmid**

**construction in this study**

| ATF2  shRNA | Sense (5’-3’) | GatccGCGGGTGACCGAAAGGATCATG  AACTTTCAAGAGAAGTTCATGATCCT  TTCGGTCACCCGTTTTTTc |
| --- | --- | --- |
|  | Antisense (5’-3’) | aattgAAAAAACGGGTGACCGAAAGGA  TCATGAACTTCTCTTGAAAGTTCATG  ATCCTTTCGGTCACCCGCg |
| Control shRNA | Sense (5’-3’) | GatccTTCTCCGAACGTGTCACGTAATT |
|  |  | CAAGAGATTACGTGACACGTTCGGAG |
|  |  | AATTTTTTg |
|  | Antisense (5’-3’) | aattcAAAAAATTCTCCGAACGTGTCAC |
|  |  | GTAATCTCTTGAATTACGTGACACGT |
|  |  | TCGGAGAAg |

**Supplemental Table 2. Sequences of primers used for qRT**-**PCR in**

**this study**

| ATF2 | Forward (5’-3’) | TGCCTGTTGCTATTCCTGC |
| --- | --- | --- |
|  | Reverse (5’-3’) | GCTCTTCTCCGACGACCACT |
| CyclinB1 | Forward (5’-3’) | TCTGGATAATGGTGAATGGACAC |
|  | Reverse (5’-3’) | CGATGTGGCATACTTGTTCTTG |
| CyclinD1 | Forward (5’-3’) | CCTGTCCTACTACCGCCTCA |
|  | Reverse (5’-3’) | TCCTCCTCTTCCTCCTCCTC |
| Snail | Forward (5’-3’) | GCTCCTTCGTCCTTCTCCTC |
|  | Reverse (5’-3’) | TGACATCTGAGTGGGTCTGG |
| [Vimentin](http://probes.pw.usda.gov/batchprimer3/batch_primers/58.32.217.78_1448850019/58.32.217.78_14488500194.html) | Forward (5’-3’) | CAGATGCGTGAAATGGAAGA |
|  | Reverse (5’-3’) | GGGTATCAACCAGAGGGAGTG |
| [E-Cadherin](http://probes.pw.usda.gov/batchprimer3/batch_primers/58.32.217.78_1448850019/58.32.217.78_14488500192.html) | Forward (5’-3’) | CAGGTCTCCTCTTGGCTCTG |
|  | Reverse (5’-3’) | GCGTGACTTTGGTGGAAAAC |
| β-Actin | Forward (5’-3’) | GTGGACATCCGCAAAGAC |
|  | Reverse (5’-3’) | AAAGGGTGTAACGCAACTA |

**Supplemental Table 3. Sequences of primers used for ChIP**-**qPCR**

**in this study**

| CyclinB1 | Forward (5’-3’) | AGAAGAGGCGGGCATTCCAGG |
| --- | --- | --- |
|  | Reverse (5’-3’) | GCCTTCTACTCCTCAAAACAC |
| CyclinD1 | Forward (5’-3’) | AGCTTTTACTGTTAAGAGGGT |
|  | Reverse (5’-3’) | ATATTATCGCAAACTTAAGCA |
| Snail | Forward (5’-3’) | GGGAGGGTTTGAGCAGAGGAG |
|  | Reverse (5’-3’) | TAGTCACTTGGTGCCGGGTGT |
| [Vimentin](http://probes.pw.usda.gov/batchprimer3/batch_primers/58.32.217.78_1448850019/58.32.217.78_14488500194.html) | Forward (5’-3’) | ACTCAGAGCATAGGCTTGTCC |
|  | Reverse (5’-3’) | TGTTTACCTTATCCCAGTTCGTT |

**Supplemental Table 4. Correlation of ATF2 expression and clinical characteristics in RCC patients**

| **Variables** | **Low ATF2 (n=103)** | **High ATF2 (n=102)** | **P-value** |
| --- | --- | --- | --- |
| **Gender** |  |  | 0.764 |
| Male | 72 | 69 |  |
| Female | 31 | 33 |  |
| **Age** |  |  | 0.771 |
| ≤60 | 68 | 65 |  |
| ＞60 | 35 | 37 |  |
| **Tumor size** |  |  | 0.007 |
| ≤4cm | 53 | 33 |  |
| ＞4cm | 50 | 69 |  |
| **Furman grade** |  |  | 0.051 |
| Ⅰ/Ⅱ | 84 | 71 |  |
| Ⅲ/Ⅵ | 19 | 31 |  |
| **TNM stage** |  |  | 0.024 |
| Ⅰ/Ⅱ | 79 | 63 |  |
| Ⅲ/Ⅵ | 24 | 39 |  |
| **Tumor thrombus** |  |  | 0.032 |
| No | 98 | 88 |  |
| Yes | 5 | 14 |  |
| **Distant metastasis** |  |  | 0.022 |
| No | 93 | 80 |  |
| Yes | 10 | 22 |  |

**Supplemental Table 5. Univariate and multivariate analyses of factors associated with overall survival in RCC patients**

| **Variable** | **Univariate** | **Multivariate** | | |
| --- | --- | --- | --- | --- |
|  |  | **Hazard Ratio** | **95% CI** | **p value** |
| **ATF2 expression** |  |  |  |  |
| High vs Low | 0.017 | 1.691 | 1.003-2.849 | 0.029^*^ |
| **Tumor size** |  |  |  |  |
| ＞4cm vs ≤4cm | 0.003 | 1.590 | 0.839-3.014 | 0.155 |
| **TNM** |  |  |  |  |
| Ⅲ/ⅥvsⅠ/Ⅱ | 0.001 | 2.325 | 1.401-3.861 | 0.001^*^ |
| **Tumor thrombus** |  |  |  |  |
| Yes vs No | 0.198 | 0.732 | 0.323-1.659 | 0.454 |
| **Distant metastasis** |  |  |  |  |
| Yes vs No | 0.002 | 1.302 | 0.628-2.700 | 0.477 |

**Supplemental Table 6. Univariate and multivariate analyses of factors associated with disease-free survival in RCC patients**

| **Variable** | **Univariate** | **Multivariate** | | |
| --- | --- | --- | --- | --- |
|  |  | **Hazard Ratio** | **95% CI** | **p value** |
| **ATF2 expression** |  |  |  |  |
| High vs Low | 0.031 | 1.905 | 1.079-3.016 | 0.045^*^ |
| **Tumor size** |  |  |  |  |
| ＞4cm vs ≤4cm | 0.002 | 1.608 | 0.915-2.827 | 0.099 |
| **TNM** |  |  |  |  |
| Ⅲ/ⅥvsⅠ/Ⅱ | 0.001 | 2.087 | 1.254-3.476 | 0.005^*^ |
| **Tumor thrombus** |  |  |  |  |
| Yes vs No | 0.223 | 0.654 | 0.304-1.410 | 0.279 |
| **Distant metastasis** |  |  |  |  |
| Yes vs No | 0.001 | 1.471 | 0.748-2.893 | 0.263 |
